# Cost-effectiveness modeling of mortality risk reduction comparing two fixed-dose combination triple therapies in moderate-to-very severe chronic obstructive pulmonary disease

**DOI:** 10.57264/cer-2025-0111

**Published:** 2025-12-15

**Authors:** Krishnali Parsekar, Shubhram Pandey, Deniz Tansey-Dwyer, Jonathan Marshall, Isabella Rustignoli, Michael Pollack, Barinder Singh, Mario Ouwens

**Affiliations:** 1 Health Economics & Payer Evidence, BioPharmaceuticals Medical, AstraZeneca, Cambridge, UK; 2 Pharmacoevidence Private Limited, Mohali, India; 3 Global Market Access & Pricing, BioPharmaceuticals Medical, AstraZeneca, Cambridge, UK; 4 Global Medical Affairs, BioPharmaceuticals Medical, AstraZeneca, Cambridge, UK; 5 Market Access, BioPharmaceuticals Medical, AstraZeneca, London, UK; 6 Respiratory Evidence Strategy, BioPharmaceuticals Medical, AstraZeneca, Wilmington, DE, USA; 7 Real World Science & Analytics, BioPharmaceuticals Medical, AstraZeneca, Gothenburg, Sweden

**Keywords:** budesonide/glycopyrronium/formoterol fumarate dihydrate, chronic obstructive pulmonary disease, cost-effectiveness, cost-effectiveness model, mortality, single-inhaler triple therapy

## Abstract

**Aim:**

Two fixed-dose combination triple therapies, budesonide/glycopyrronium/formoterol fumarate dihydrate (BGF) 320/14.4/10 μg and fluticasone furoate/umeclidinium/vilanterol (FF/UMEC/VI) 100/62.5/25 μg, have been shown to reduce all-cause mortality in phase III trials. A recent matching-adjusted indirect comparison showed greater mortality reduction with BGF versus FF/UMEC/VI. However, the comparative cost-effectiveness of these treatments remains unknown. This study aimed to use a cost-effectiveness model to compare BGF with FF/UMEC/VI in patients with moderate-to-very severe chronic obstructive pulmonary disease (COPD) who are eligible for triple therapy from a UK third-party payer perspective.

**Materials & methods:**

A cohort-based semi-Markov model was used with the natural progression of COPD defined by four lung function levels. Input parameters included participant characteristics and clinical efficacy parameters derived from the ETHOS and KRONOS trials, the UK general population and published literature. Unit costs were obtained from the UK National Health System Schedule of Reference Costs (2021/2022), the Personal Social Services Research Unit (2022) and published literature. Outputs included an incremental cost-effectiveness ratio, costs, quality-adjusted life years and life years at time horizons of 1 and 5 years. Model inputs and assumptions were subject to deterministic and probabilistic sensitivity and scenario analyses.

**Results:**

At both 1- and 5-year time horizons, BGF was less costly (-£7.24 and -£23.03 per patient) and more effective (0.002 and 0.21 quality-adjusted life years per patient) versus FF/UMEC/VI, respectively. Due to a reduced mortality rate, more patients remained on BGF treatment than on FF/UMEC/VI, which induced higher treatment-related costs; however, the latter was offset by decreased end-of-life costs, as BGF avoided more deaths.

**Conclusion:**

BGF may improve health outcomes and reduce healthcare costs compared with FF/UMEC/VI in patients with moderate-to-very severe COPD in a UK setting.

## Plain language summary: comparing costs & benefits of two treatments for COPD

**What is this article about?:**

Chronic obstructive pulmonary disease (COPD) is a serious lung disease that makes breathing difficult and worsens over time. COPD greatly impacts healthcare systems and a person’s quality of life. This study examined two inhaler treatments for COPD: budesonide/glycopyrronium/formoterol fumarate dihydrate (BGF) and fluticasone furoate/umeclidinium/vilanterol (FF/UMEC/VI). We aimed to understand which treatment was more effective at lowering healthcare costs, improving quality of life and reducing death in a UK setting.

**What methodology/protocol is described?:**

COPD guidelines recognize that BGF and FF/UMEC/VI lower the risk of death, but a comparison of costs is lacking. A cost-effectiveness model was developed using phase III clinical trials with BGF and a published matching-adjusted indirect treatment comparison study of BGF and FF/UMEC/VI. The model evaluated the total healthcare costs and health benefits of BGF and FF/UMEC/VI inhaler treatments over 1- and 5-year periods in COPD that worsened over time.

**What were the results?:**

The model showed that BGF was less costly and more effective compared with FF/UMEC/VI because it reduced deaths, which led to lower end-of-life care costs, even when taking BGF for a longer period.

**Why is this important?:**

This study suggests that BGF not only improves patient health outcomes but could also reduce healthcare costs within the duration of 1 to 5 years. The findings support the use of BGF as a cost-effective treatment for patients with COPD, which may lead to better management of this disease.

Chronic obstructive pulmonary disease (COPD) is a heterogeneous lung disease characterized by persistent respiratory symptoms due to restricted airflow and breathing problems [1]. Respiratory symptoms associated with COPD may substantially impact healthcare burden and may lead to a decline in health-related quality of life (HRQoL) [2]. Patients with COPD are more likely to develop other serious health conditions, such as lung infections, lung cancer and cardiovascular diseases, which further complicates disease management and impacts patient outcomes [3]. Cardiopulmonary complications are a leading cause of death in patients with COPD [4,5]. Moreover, COPD exacerbations increase the likelihood of future cardiovascular events and elevate the risk of mortality from all causes, as well as from COPD and cardiovascular conditions [6–8]. The progressive nature of COPD and its associated comorbidities necessitate comprehensive treatment strategies. Globally, COPD is responsible for approximately 3 million deaths annually, making it the third leading cause of death worldwide (excluding COVID-19) [9]. The high mortality underscores the urgent need for effective therapeutic interventions.

Patients with COPD are frequently hospitalized, have increased medication needs and have frequent recurrent medical visits, which all contribute to elevated healthcare resource utilization (HCRU) and costs [2]. Furthermore, COPD is a leading cause of work absence and premature retirement, resulting in considerable economic implications for both individuals and healthcare systems [10,11]. Healthcare system costs associated with COPD currently account for approximately £2 billion annually in the UK [12], €39 billion annually in the European Union [13] and $40 billion annually in the US [13,14]. These costs contribute to an estimated global economic burden that exceeds $4 trillion US dollars (€3.7 trillion Euros) from 2020 to 2050 [9].

Recent advancements in treatment options have focused on fixed-dose combination triple therapy. Notably, two large phase III studies, ETHOS (NCT02465567) and IMPACT (NCT02164513), demonstrated that fixed-dose combination triple therapy (budesonide/glycopyrronium/formoterol fumarate dihydrate [BGF] 320/14.4/10 μg in ETHOS [15] and fluticasone furoate/umeclidinium/vilanterol [FF/UMEC/VI] 100/62.5/25 μg in IMPACT [16]) reduced all-cause mortality risk compared with dual therapy in patients with COPD [11].

A recent matching-adjusted indirect comparison (MAIC) based on the ETHOS and IMPACT studies provided further evidence of the efficacy of fixed-dose combination triple therapy, particularly on mortality. The MAIC reported a significantly greater reduction in mortality risk with BGF compared with FF/UMEC/VI in patients with moderate-to-very severe COPD (hazard ratio [95% confidence interval]: 0.61 [0.38, 0.95], p = 0.030) [17]. Building on these findings, the objective of this cost-effectiveness evaluation was to assess the impact of BGF on costs and mortality compared with FF/UMEC/VI in patients with moderate-to-very severe COPD who are eligible for triple therapy from the perspective of a UK third-party payer over different time horizons.

## Materials & methods

### Setting & patient population

The model population had moderate-to-very severe COPD, were symptomatic and had a history of exacerbations. Clinical data were used from the modified intention-to-treat population (patients who underwent randomization, received trial treatment and had post-randomization data obtained before discontinuation of treatment) of patients in the ETHOS study who had an established clinical history of COPD [18], from patients in the KRONOS (NCT02497001) study with no recent history of COPD exacerbation (post 1-year, if no exacerbations) [19] and the MAIC study between patients from the ETHOS and IMPACT trials [17,20]. MAIC studies were chosen as these studies attempt to reduce bias in treatment comparisons by matching individual patient-level data from clinical trials of one treatment to aggregate data reported for comparator trials. Because the eligibility criteria differed slightly between the ETHOS and IMPACT trials, slight differences were observed for sex, race, BMI, COPD severity and exacerbation history [17]. One-year and 5-year time horizons were considered for the base case. Annual discount rates were applied for costs, quality-adjusted life years (QALYs) and life years. Discount rates were 3.5% for costs and outcomes, as aligned with National Institute for Health and Care Excellence guidance [21].

### Model structure

**Figure 1.**
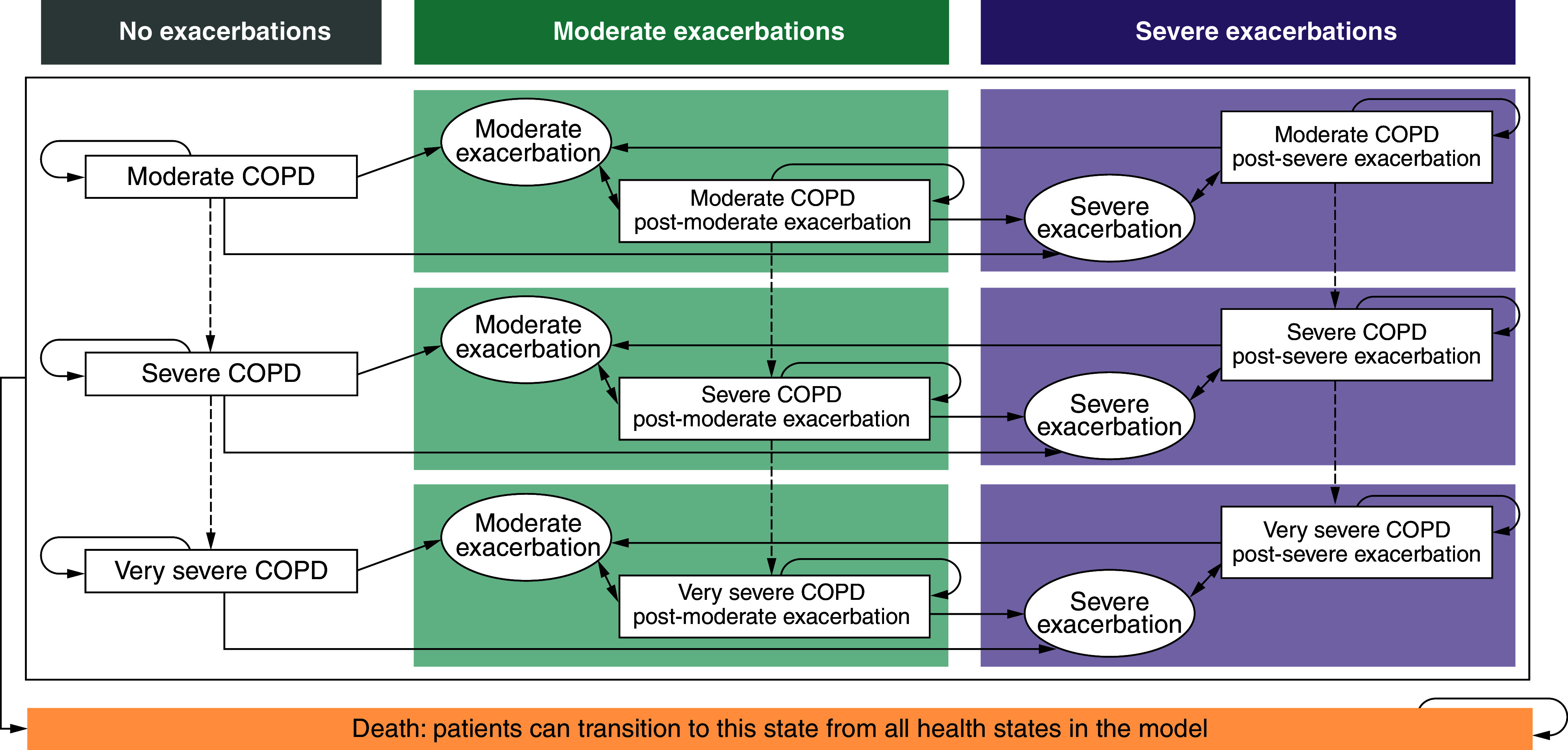
Semi-Markov model^a^. ^a^Moderate COPD was defined as having a FEV_1_ between 50% and <80% of the predicted normal, severe COPD was defined has having a FEV_1_ between 30% and <50% of the predicted normal and very severe COPD was defined as a FEV_1_ <30% of the predicted normal. Moderate COPD exacerbations were defined as those requiring use of systemic glucocorticoids, antibiotics or both for ≥3 days. Severe COPD exacerbations were defined as those resulting in hospitalization or death. COPD: Chronic obstructive pulmonary disease; FEV_1_: Forced expiratory volume in 1 s.

This cohort-based study used a semi-Markov model, consisting of four COPD severity levels based upon Global Initiative for Chronic Obstructive Lung Disease (GOLD) grades and severity defined by lung function, that reflects stages during the natural progression of COPD (Figure 1) [22]. The model was developed in Microsoft^®^ Excel 2010. Participants remain within the same severity, transition to a more severe COPD state, experience exacerbations or die. Health-state–specific costs (e.g., disease management), treatment-related costs and utilities were assigned to each COPD severity level.

COPD severity levels were defined as follows [22]:

- GOLD 2: moderate COPD (forced expiratory volume in 1 second [FEV_1_]% predicted between 50% and <80%)
- GOLD 3: severe COPD (FEV_1_% predicted between 30% and <50%)
- GOLD 4: very severe COPD (FEV_1_% predicted <30%)

Health states were also differentiated by the history of exacerbations since the beginning of the model simulation (post-severe exacerbation, post-moderate exacerbation or no exacerbation). Each lung function state was replicated three times to distinguish patients who had experienced severe exacerbations, moderate exacerbations or no exacerbations.

‘Tunnel-states’ of one model cycle were defined as sub-states of each COPD severity level that correspond to moderate or severe exacerbations. From the tunnel state, patients transition to either the applicable post-exacerbation COPD severity level or death state. A moderate exacerbation was defined as requiring treatment with systemic corticosteroids, antibiotics or both for ≥3 days. A severe exacerbation was defined as requiring hospitalization or whose exacerbation resulted in death. The death state was an absorbing health state, where patients could transition from other health states throughout the time horizon, associated with end-of-life cost (30 days prior to event) and a utility of 0.

The patient cohort entered the model starting in the no exacerbation health state and was distributed across COPD severity levels of airflow limitation based on the ETHOS study population at baseline (Supplementary Table 1) [23]. The distribution of patients included moderate COPD, 28.5%; severe COPD, 60.6%; and very severe COPD, 10.9% (Supplementary Table 2). Patients received either BGF or FF/UMEC/VI for the treatment of COPD. During each monthly cycle, patients could transition to a more severe COPD severity level, transition to a moderate or severe exacerbation health state – if considered very severe at the start of each cycle, remain within the current COPD severity level or die. As COPD is a progressive disease, patients could not transition to a mild COPD severity level, nor could they experience an improvement in lung function and transition back to a less severe COPD severity level [23]. Exacerbation states were treated as discrete events lasting 1 monthly cycle, and patients could only experience up to one exacerbation per cycle but were at risk of further exacerbations in subsequent monthly cycles. Patients could experience moderate or severe exacerbations in each model cycle based on their COPD severity level. Weekly exacerbation rates were converted to monthly probabilities to reflect the model cycle length and remained constant over time. Using data from KRONOS and ETHOS, increased (or decreased) rates of further exacerbations could be applied in post-exacerbation health states (compared with the exacerbation-free health states) [19,23].

The model perspective was based on the third-party payer, National Health Service (NHS) in England, and included all direct costs and health effects relevant to the NHS.

### Data sources

#### Lung function/airflow limitation

The BGF COPD severity level transition probabilities were obtained from the ETHOS and KRONOS trials [23,24]. Transition probabilities up to cycle 12 were obtained from the ETHOS trial. From cycle 12 (52 weeks) and until the time horizon, for patients who did not experience an exacerbation, transitions from KRONOS were applied with the purpose of representing a population with no recent exacerbation history. The 52-week probabilities were converted to monthly probabilities to align with the model’s 1-month cycle length (Table 1). At 52 weeks, the network meta-analysis (NMA) demonstrated comparable improvement in trough FEV_1_ for BGF compared with FF/UMEC/VI; therefore, the same transition probabilities were considered between the COPD severity levels for both treatments [11].

**Table 1.** Chronic obstructive pulmonary disease severity level transitions.

COPD severity level transition	Monthly probability	Relative risk vs BGF	Ref.
	BGF	FF/UMEC/VI	
No recent exacerbation history			
Moderate COPD to severe COPD	1.13%	1.00^‡^	
Severe COPD to very severe COPD	0.87%	1.00^‡^	
Source	Kronos payer analysis plan	Assumption	[24]
Post-moderate exacerbation			
Moderate COPD to severe COPD	2.27%^†^	1.00^‡^	
Severe COPD to very severe COPD	0.60%^§^	1.00^‡^	
Source	de Nigris 2022 (ETHOS)	Bourdin NMA	[11,23]
Post-severe exacerbation			
Moderate COPD to severe COPD	2.27%^†^	1.00^‡^	
Severe COPD to very severe COPD	0.60%^§^	1.00^‡^	
Source	de Nigris 2022 (ETHOS)	Bourdin NMA	[11,23]

† [1-EXP(1)^∧^(-(-LN(1-“probability of transitioning from moderate to severe and very severe COPD at week 52 for BGF”)/((12*52)/52))) = 1-EXP(1)^∧^(-(-LN(1–24.10%)/((12*52)/52))) = 2.27%].

‡ It was assumed that the relative risks are equal to 1 due to lack of data (transition probabilities equal to BGF).

§ [1-EXP(1)^∧^(-(-LN(1-“probability of transitioning from severe to very severe COPD at week 52 for BGF”)/((12*52)/52))) = 1-EXP(1)^∧^(-(-LN(1–7%)/((12*52)/52))) = 0.60%].

BGF: Budesonide/glycopyrronium/formoterol fumarate dihydrate; COPD: Chronic obstructive pulmonary disease; FF/UMEC/VI: Fluticasone furoate/umeclidinium/vilanterol; NMA: Network meta-analysis.

#### Exacerbations

Moderate and severe exacerbation rates were assessed for the overall patient population and stratified by lung function severity at baseline. The 52-week probabilities were converted to monthly probabilities. To derive the transition probabilities for FF/UMEC/VI, relative risk was applied from the NMA versus BGF (Supplementary Table 3) [11]. Lung function deterioration and exacerbations were assumed constant over the full treatment duration for both treatments.

#### Treatment discontinuation

The treatment discontinuation risk for BGF was obtained from the ETHOS publication for the previous cost-effectiveness analysis [23]; for the comparator, the risk of discontinuation was obtained from published literature for FF/UMEC/VI (Supplementary Table 4) [20]. These rates were applied for up to 1 year. Subsequent treatment efficacy was assumed to be equal to initial treatment (relative risks and rate ratios were equal to 1). The monthly risk of subsequent treatment discontinuation for all treatments was set at 1% per cycle. After treatment discontinuation, patients were assumed to receive subsequent treatment with BGF plus roflumilast as described by key external experts and supported by GOLD 2024 recommendations [13,23].

#### Mortality

The model had the option of choosing Kaplan–Meier (KM) curve data (used in base case analysis) or constant probability based on overall events in each respective trial. For the KM curve option, the MAIC-adjusted KM curves were digitized to extract the cumulative mortality data, which was further split up into moderate COPD, severe COPD and very severe COPD health states based on COPD-related mortality relative risks (Table 2). Extrapolation after 12 months was based on the ratio of instantaneous hazards in month 12. Details of the digitization approach, survival models tested, goodness-of-fit assessments and rationale for the selected extrapolation have been previously described [17]. For the constant probability option, a constant probability of death was assumed for the first 12 months, with the yearly probability of death adjusted to a monthly probability to reflect the model cycle length. The monthly probability of death was calculated to be 0.118% for BGF and 0.193% for FF/UMEC/VI.

**Table 2.** Mortality relative risks derived from the published literature.

COPD severity level–related mortality	Relative risk	Author, year	Ref.
Moderate COPD no exacerbation-related mortality^†^	1.40	Shavelle 2009	[35]
Severe COPD no exacerbation-related mortality^†^	2.60	Shavelle 2009	[35]
Very severe COPD no exacerbation-related mortality^†^	2.60	Shavelle 2009	[35]
All COPD severity levels after moderate exacerbation^‡^	1.00	Assumption	
All COPD severity levels after severe exacerbation^‡^	1.00	Assumption	

† Relative risk vs annual general population mortality.

‡ Relative risk vs probability of death for health states without exacerbation history.

COPD: Chronic obstructive pulmonary disease.

Mortality related to severe exacerbations was captured separately. In the first year, the input was set to 0% to avoid double-counting from overall mortality rates in the clinical trial. Beyond the first year, 12% severe exacerbation–related mortality was used (Table 3). Given the assumptions described above and limited data availability to the trial duration (52 weeks), several scenario analyses were explored (described below).

**Table 3.** Exacerbation-related mortality.

Exacerbation-related mortality	Event probability	Author, year	Ref.
Severe exacerbation-related mortality	12.0%^†^	NICE, Royal College of Physicians	[36–38]

† This value (12%) was extracted from the roflumilast NICE submission as the preferred post-hospitalization mortality input for the model based on the evidence review group assessment, as the most recent (2014) UK-specific estimate for post-hospitalization mortality from the UK National COPD Audit. This value reflects the 90-day post-hospitalization mortality estimate for patients who have experienced a severe exacerbation. In the current model, the effect of severe exacerbation on mortality can only be applied in 1 cycle, which equals 1 month.

The severe exacerbation-related mortality event probability of 12.0% was derived as follows: 0.043+(1-0.043)×8%=11.98%∼12%.

COPD: Chronic obstructive pulmonary disease; NICE: National Institute for Health and Care Excellence.

The probabilities for all-cause mortality for the general population were derived from age- and gender-specific mortality rates from UK lifetables published by the Office of National Statistics [25]. According to the published data, mortality rates in males were generally higher than mortality rates in females, therefore this difference was accounted for in the model as follows [26]:

(Equation 1) $$\begin{array}{ll} Proportion\;of\;people\;alive\;at\;age\;x & =\left(1-rate\;of\;dying\;at\;age\;\left(x-1\right)\right)\\ & \times \;Proportion\;of\;people\;alive\;at\;age\;x-1 \end{array}$$

(Equation 2) $$Proportion\;female\;at\;age\;x=\;\frac{\left(p1\times p2\right)}{\left(p1\times p2+\left(1-p2\right)\times p3\right)}$$

where

$$\begin{array}{l} p1=Proportion\;of\;females\;alive\;at\;age\;x\\ p2=Proportion\;of\;females\;at\;model\;start\\ p3=Proportion\;of\;males\;alive\;at\;age\;x \end{array}$$

The general population mortality at a specific age was calculated as the weighted average of the proportion of males and females at that age group as follows:

(Equation 3) General pop mortality=r1×p+r2×(1-p)

$$\begin{array}{l} p=\;Proportion\;of\;females\;at\;that\;age\;group\\ r1=\;Mortality\;rate\;of\;females\;at\;that\;age\\ r2=\;Mortality\;rate\;of\;males\;at\;that\;age \end{array}$$

The general population mortality was adjusted beyond 1 year because mortality data from the trial were used for the first 12 months and it was assumed that these data already accounted for general population mortality.

Mortality inputs were derived from the published MAIC of ETHOS and IMPACT, and matched key baseline covariates between trials and reported balance diagnostics, effective sample sizes and residual imbalances [17].

#### Adverse events

Data for treatment-related adverse events (AEs) were obtained from the ETHOS trial [23]. The relative risk of AEs for fixed-dose combination triple therapies versus BGF were extracted from the NMA (Supplementary Table 5) [11]. Due to the low incidence of serious AEs in ETHOS, only the incidence and relative risks of mild, moderate and severe AEs were included in this model, resulting in a conservative evaluation. Costs and disutilities associated with AEs were applied at the beginning of the model, ensuring the full impact of AEs was captured.

#### Utility & HRQoL

Severity level–related utilities were 0.79 for moderate COPD, 0.76 for severe COPD and 0.72 for very severe COPD (Supplementary Table 6) [23]. Utility decrements were also applied per exacerbation event: 0.055 for moderate exacerbations [27] and 0.090 for severe exacerbations [28]. No additional AE-related disutility was considered as it was assumed these effects were captured in the severity and exacerbation disutilities. An overview of the utility decrements is presented in Supplementary Table 7.

#### HCRU

COPD-related HCRU incorporated in the economic model included disease management costs (e.g., lung-function–related and exacerbation-related) and treatment-specific costs such as drug acquisition, rescue medication, treatment-related AEs, subsequent treatment and end-of-life costs. Resource use was based on ETHOS and published literature [29].

#### Costs

Unit costs were obtained from targeted literature searches including the following UK agencies: NHS reference costs, Personal Social Services Research Unit (PSSRU) [30,31] and the British National Formulary [32] for drug acquisition costs.

#### Lung-function–related cost

Monthly disease management costs by COPD severity level were obtained from the resource consumption from ETHOS data [23], and unit cost per resource was collected from published literature, including NHS reference costs or PSSRU (Supplementary Table 8) [30,31].

#### Exacerbation-related cost

Moderate exacerbation costs were £53.23 per event, and severe exacerbation costs were £3667.35 per event. These costs were derived from a previous cost-effectiveness model [23].

#### Treatment-specific cost

Treatment costs were separated into acquisition costs (Supplementary Table 9), rescue medication costs (Supplementary Table 10), treatment-related AE costs (Supplementary Table 11) and subsequent treatment costs associated with treatment discontinuation or escalation (Supplementary Table 12).

#### End-of-life cost

In addition to exacerbation costs, end-of-life/terminal care costs were applied as a one-time cost to all patients entering the death health state and were calculated from the last 30 days of HCRU based on published real-world evidence in the UK [29]. In addition to exacerbations costs, in the base case it was assumed that all patients died in the hospital with an associated cost of £4028 [29]. Scenario analysis explored different settings during end-of-life.

#### Uncertainty

Deterministic sensitivity analysis examined the impact of varying a parameter by ±20% of its deterministic value as part of the one-way sensitivity analysis.

#### Scenario analysis

Several scenarios were explored to assess the long-term impact of mortality extrapolation as well as setting during end-of-life. This included the waning effect on mortality scenario, mortality curve using log-normal distribution and setting during end-of-life. Further details are included in the Supplementary Methods & Supplementary Figure 1.

## Results

### Base case results

The evaluation revealed that BGF is a less costly and more effective (dominant) treatment strategy compared with FF/UMEC/VI at both 1- and 5-year time horizons (Table 4). Specifically, BGF was associated with a gain in life years of 0.004 at 1 year and 0.31 at 5 years. Additionally, BGF demonstrated an increase in QALYs of 0.002 at 1 year and 0.21 at 5 years. From an economic perspective, BGF was associated with reduced per patient total costs of -£7.24 over 1 year and -£23.03 over 5 years (Table 4). However, treatment-related costs were higher with BGF versus FF/UMEC/VI due to the lower mortality rate associated with BGF (Table 5).

**Table 4.** Dominance analysis for BGF versus FF/UMEC/VI.

Outcome	BGF vs FF/UMEC/VI	
	1 year	5 year
ICUR (cost per QALY gained)	Less costly and more effective^†^	Less costly and more effective^†^
ICER (cost per LY gained)	Less costly and more effective^†^	Less costly and more effective^†^
Incremental costs per mortality avoided	Less costly and more effective^†^	Less costly and more effective^†^
Incremental net benefits based on willingness-to-pay threshold of £20,000	54.03	4310.79
Total incremental costs, £	-7.24	-23.03
Incremental disease management costs, £	15.20	313.60
Incremental treatment-specific costs, £	-3.65	73.66
Incremental subsequent treatment costs, £	10.15	181.90
Incremental end-of-life costs, £	-28.94	-592.19
Total incremental QALYs	0.002	0.21
Total incremental LYs	0.004	0.31
Total incremental exacerbations	0.007	0.33
Total incremental deaths avoided	0.88%	16.10%

† Dominant.

BGF: Budesonide/glycopyrronium/formoterol fumarate dihydrate; FF/UMEC/VI: Fluticasone furoate/umeclidinium/vilanterol; ICER: Incremental cost-effectiveness ratio; ICUR: Incremental cost-utility ratio; LY: Life year; QALY: Quality-adjusted life year.

**Table 5.** Total costs and outcomes at the 5-year time horizon.

Outcome	BGF	FF/UMEC/VI
Total costs, £	7866.0	7889.10
Disease management costs	3523.50	3209.90
Treatment-specific costs before discontinuation	2563.50	2489.90
Subsequent treatment costs	1342.40	1160.50
End of life costs	436.60	1028.78
Total QALYs	3.11	2.90
Total LYs	4.44	4.12

BGF: Budesonide/glycopyrronium/formoterol fumarate dihydrate; FF/UMEC/VI: Fluticasone furoate/umeclidinium/vilanterol; LY: life year; QALY: Quality-adjusted life year.

### Sensitivity analysis

**Figure 2.**
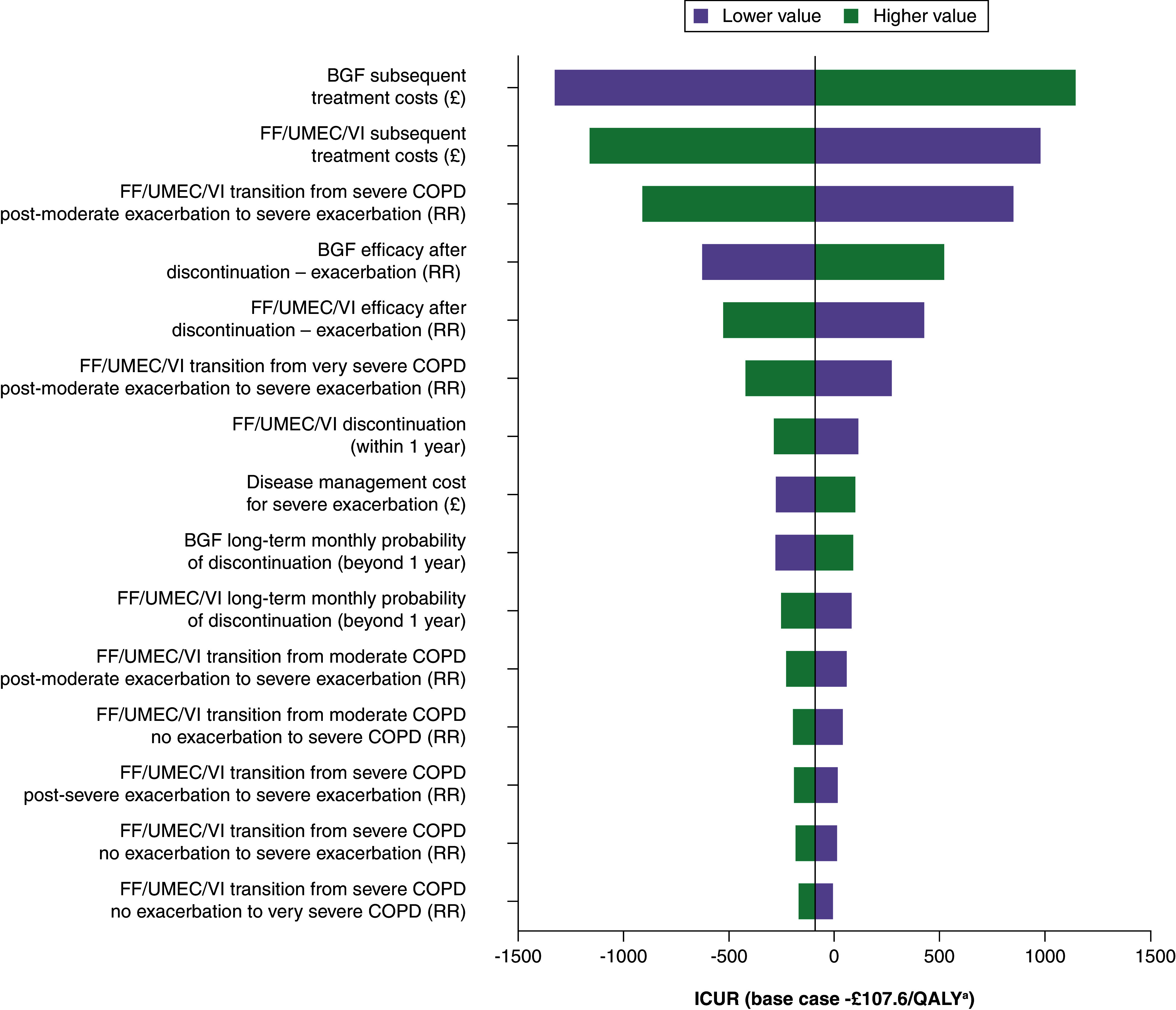
Key drivers of cost-effectiveness. ^a^23.03/0.214. BGF: Budesonide/glycopyrronium/formoterol fumarate dihydrate; COPD: Chronic obstructive pulmonary disease; FF/UMEC/VI: Fluticasone furoate/umeclidinium/vilanterol; ICUR: Incremental cost-utility ratio; QALY: Quality-adjusted life year; RR: Relative risk.

Deterministic sensitivity analysis identified key drivers, including subsequent treatment costs for both BGF and FF/UMEC/VI, severe exacerbation rates at severe and very severe COPD severity levels for FF/UMEC/VI and efficacy on exacerbation rates post-discontinuation for both BGF and FF/UMEC/VI (Figure 2).

### Scenario analysis

BGF remains cost-effective compared with FF/UMEC/VI over a 5-year time horizon, even when considering a diminishing treatment effect over time (Table 6 & Supplementary Table 13). Similar results were observed when fitting log-normal distribution on a MAIC-adjusted KM curve and different distribution of care settings during end-of-life (Supplementary Tables 14 & 15 & Supplementary Figure 2A & B).

**Table 6.** 5-year time horizon scenario analyses for BGF versus FF/UMEC/VI.

Outcome	Waning effect^†^	Log-normal mortality distribution	End of life setting^‡^
ICUR (cost per QALY gained), £	818.79	370.22	912.16
ICER (cost per LY gained), £	565.82	255.20	631.17
Incremental costs per mortality avoided, £	1588.27	538.20	1214.68
Incremental net benefits based on willingness-to-pay threshold of £20,000	2802.20	2419.94	4092.21
Total incremental costs, £	119.62	45.64	195.56
Incremental disease management costs	228.32	202.75	313.60
Incremental treatment-specific costs	42.06	29.26	73.66
Incremental subsequent treatment costs	138.41	128.12	181.90
Incremental end-of-life costs	-289.17	-314.49	-373.60
Total incremental QALYs	0.15	0.12	0.21
Total incremental LYs	0.21	0.18	0.31

† A waning effect on mortality was applied after 1 year such that the treatment effect of BGF on mortality had a linear reduction and was comparable to FF/UMEC/VI at the 5-year time horizon.

‡ In contrast to the base-case scenario, which assumed all deaths are experienced in the hospital setting, this analysis applied a distribution of care setting from the INteractive Health Atlas of Lung conditions in England (INHALE) Office for Health Improvement and Disparities 2023.

BGF: Budesonide/glycopyrronium/formoterol fumarate dihydrate; FF/UMEC/VI: Fluticasone furoate/umeclidinium/vilanterol; ICER: Incremental cost-effectiveness ratio; ICUR: Incremental cost-utility ratio; LY: Life year; QALY: Quality-adjusted life year.

## Discussion

The aim of this evaluation was to assess the cost-effectiveness of fixed-dose combination triple therapy with BGF versus FF/UMEC/VI in patients with moderate-to-very severe COPD in the UK. The key finding was that BGF was more effective and less costly than FF/UMEC/VI at 1- and 5-year time horizons, respectively. BGF remained cost-effective at the UK-adopted willingness-to-pay threshold (£20,000/QALY gained) when exploring different scenarios of long-term mortality extrapolation and end-of-life settings.

The cost-effectiveness model showed that treatment-related costs for BGF may be higher than those for FF/UMEC/VI because patients live longer with BGF, which results in more patients remaining on BGF for a longer period. However, the BGF treatment–related costs were offset by decreased end-of-life costs within both 1- and 5-year time horizons. Given that real-world evidence from the UK demonstrates that end-of-life costs in the last 30 days are high [29], particularly when compared with the evaluated costs associated with BGF, the economic benefit of extending patient survival through effective treatment may lead to cost savings. Nevertheless, cost savings will vary due to differences in healthcare delivery models, unit costs and reimbursement structures in other countries. Therefore, future country-specific modeling studies are warranted to validate and quantify these effects in different healthcare environments.

The model incorporated data from a recently published MAIC comparing BGF with FF/UMEC/VI based on the ETHOS and IMPACT studies [15–17]. As with an earlier study comparing BGF with dual therapies, this evaluation continues to support BGF as a cost-effective option to treat COPD. By utilizing these diverse evidence sources, the model aimed to provide a comprehensive assessment of cost-effectiveness in the absence of direct comparative studies.

This cost-effectiveness model was able to explore a variety of scenarios for extrapolating mortality risk beyond 1 year. Scenarios included waning treatment effects on mortality, log-normal mortality distribution, different distributions for end-of-life settings and associated costs for BGF versus FF/UMEC/VI. Across these scenarios, BGF remained cost-effective, with an incremental cost-effectiveness ratio well below the UK threshold of £20,000 per QALY [33]. When projected to a population level, for every 1000 patients treated with BGF, the model estimates that over 1 year: 8 deaths would be avoided, 2.3 QALYs would be gained and £7243 in healthcare costs would be saved. Over 5 years, this translates to 161 deaths avoided, 214.4 QALYs gained, and £23,027 in cost savings. These findings reinforce the clinical value of mortality risk reduction associated with fixed-dose triple combinations, as recognized in the GOLD guidelines [13], while also generating health system cost savings.

There were several limitations to this evaluation. Real-world studies could offer more robust long-term estimates than the several scenarios run for mortality based on 1-year data. Discontinuation rates came from each respective trial, with a 1% monthly risk assumed beyond year 2 due to limited long-term data. The analyses used inputs from a MAIC without a head-to-head trial, which may have limitations as noted in the primary MAIC mortality analyses of BGF versus FF/UMEC/VI [17]. This evaluation is confined to a time horizon of up to 5 years and is acknowledged as a study limitation. From a policy perspective, a 4- to 5-year time horizon may be relevant, as maintenance treatment strategies for patients with COPD will hopefully change in the future [34].

## Conclusion

This cost-effectiveness model demonstrated that, when compared with FF/UMEC/VI, BGF would improve health outcomes and reduce healthcare costs in patients with moderate-to-very severe COPD in a UK setting. The model’s findings suggest that BGF, compared with FF/UMEC/VI, is a cost-saving treatment option that can provide significant health benefits while also offering economic advantages by reducing overall healthcare expenditures.

## Summary points

- The article examines the cost-effectiveness of two fixed-dose combination triple therapies for moderate-to-very severe chronic obstructive pulmonary disease (COPD): budesonide/glycopyrronium/formoterol fumarate dihydrate (BGF) and fluticasone furoate/umeclidinium/vilanterol (FF/UMEC/VI).
- COPD is a leading cause of death worldwide, contributing to substantial clinical and socioeconomic challenges.
- The study used a cohort-based semi-Markov model to analyze the cost-effectiveness of BGF compared with FF/UMEC/VI over 1-year and 5-year periods.
- Results indicated that BGF is less costly and improved quality-adjusted life years more than FF/UMEC/VI, with cost savings of £7.24 and £23.03 per person and an increase in quality-adjusted life years per person of 0.002 and 0.21 over 1-year and 5-year periods, respectively.
- BGF treatment led to higher treatment-related costs due to longer patient survival but was offset by decreased end-of-life costs, resulting in overall cost savings.
- BGF showed a greater reduction in mortality risk compared with FF/UMEC/VI, with a 16.10% reduction in deaths over 5 years.
- The study supports BGF as a cost-effective treatment for COPD, improving health outcomes and reducing healthcare costs in the UK.
- This evaluation was based on data from clinical trials and real-world evidence, aiming to provide a comprehensive assessment in the absence of direct comparative studies.

## Supplementary Material






