# Supplementary material for: Cost-effectiveness modeling of mortality risk reduction comparing two fixed-dose combination triple therapies in moderate-to-very severe chronic obstructive pulmonary disease
Source: J Comp Eff Res. 2025 Dec 15;15(1):e250111. doi: 10.57264/cer-2025-0111 (PMC12711099; doi:10.57264/cer-2025-0111)
Supplement: Supplementary file 3 [file cer-15-250111-s3.docx]

**Supplementary materials**

**Supplementary methods:**

**Waning effect on mortality:** In this scenario, linear waning effect on mortality was applied in a way that starting after 1 year, the treatment effect on mortality for BGF had linear reduction which was comparable to FF/UMEC/VI at 5 years. The model assumed that the treatment effect wanes linearly in between the specified time point i.e. starting time-point of treatment waning, which was assumed as 1-year in the base case and the end-timepoint which was assumed as time-horizon in the base case. At the starting point of treatment waning, the hazard was equal to the BGF, and it increased linearly up to the last time point where the hazard of BGF was equal to the hazard of FF/UMEC/VI. A depictive example of linear waning is shown in Supplementary Figure S1.

**Mortality curve using log-normal distribution**: In this scenario, rather than using the MAIC-adjusted KM curves directly, statistical distributions such as Exponential, Weibull, Log-normal, Log-logistic, and generalized gamma were fitted on MAIC-adjusted KM curves and best-fitted distribution was selected to derive the mortality curve. Log-normal distribution showed the best fit for data according to goodness of fit statistic (AIC, BIC) and visual inspection.

**Setting during end-of-life**: While the base case considered that all deaths were experienced in the hospital setting, in the scenario analysis, a distribution of care settings was applied. The proportion of patients requiring different settings i.e. hospital, hospice, and home care was derived from the UK government website, 2021 with values equivalent to 54.7%, 31.3%, and 11.1%, respectively.[1]

**Supplementary tables:**

**Supplementary Table 1** Model input population characteristics

| Characteristics | ETHOS modified intention-to-treat population[2] |
| --- | --- |
| Mean (SD) age | 64.6 (7.6) |
| Proportion women (%) | 41 |

**Abbreviation:** SD, standard deviation.

**Supplementary Table 2** Patient distribution on the COPD severity levels at the beginning of the time horizon

| COPD severity levels | ETHOS modified intention-to-treat population[2] |
| --- | --- |
| Moderate COPD no exacerbations | 28.70% |
| Severe COPD no exacerbations | 61.10% |
| Very severe COPD no exacerbations | 10.20% |

**Abbreviation:** COPD, chronic obstructive pulmonary disease.

**Supplementary Table 3** Exacerbation rates and relative risks

| Transitions from COPD severity levels to exacerbation states | Adjusted^a^ monthly rates | Relative risks (versus BGF) |
| --- | --- | --- |
|  | BGF | FF/UMEC/VI |
| No recent exacerbation history | | |
| Moderate COPD exacerbations to moderate exacerbation | 0.028 | 1.00 |
| Moderate COPD exacerbations to severe exacerbation | 0.001 | 1.00 |
| Severe COPD exacerbations to moderate exacerbation | 0.038 | 1.00 |
| Severe COPD exacerbations to severe exacerbation | 0.008 | 1.00 |
| Very severe COPD exacerbations to moderate exacerbation | 0.013 | 1.00 |
| Very severe COPD exacerbations to severe exacerbation | 0.006 | 1.00 |
| Source | Kronos payer analysis plan[3] | Assumption |
| Post-moderate exacerbation | | |
| Moderate COPD exacerbations to moderate exacerbation | 0.066 | 1.00 |
| Moderate COPD exacerbations to severe exacerbation | 0.007 | 0.97 |
| Severe COPD exacerbations to moderate exacerbation | 0.077 | 1.00 |
| Severe COPD exacerbations to severe exacerbation | 0.013 | 0.97 |
| Very severe COPD exacerbations to moderate exacerbation | 0.088 | 1.00 |
| Very severe COPD exacerbations to severe exacerbation | 0.017 | 0.97 |
| Source | de Nigris 2022 (ETHOS) [2] | Bourdin NMA[4] |
| Post-severe exacerbation | | |
| Moderate COPD exacerbations to moderate exacerbation | 0.066 | 1.00 |
| Moderate COPD exacerbations to severe exacerbation | 0.007 | 0.97 |
| Severe COPD exacerbations to moderate exacerbation | 0.077 | 1.00 |
| Severe COPD exacerbations to severe exacerbation | 0.013 | 0.97 |
| Very severe COPD exacerbations to moderate exacerbation | 0.088 | 1.00 |
| Very severe COPD exacerbations to severe exacerbation | 0.017 | 0.97 |
| Source | de Nigris 2022 (ETHOS) [2] | Bourdin NMA[4] |

**Notes:** ^a^The adjusted monthly rates were calculated based on the adjusted annual rates [-LN(1-(1-EXP(1)^(-"adjusted annual exacerbation rate for BGF"*(1/12))))] (eg, for BGF for moderate exacerbation on moderate COPD state: -LN(1-(1-EXP(1)^(-0.79*(1/12)))) = 0.066). Adjusted means that treatments were compared adjusting for baseline post-bronchodilator percent predicted COPD and baseline eosinophil count as continuous covariates and baseline COPD exacerbation history (0, 1, ≥2), and inhaled corticosteroid use at screening (yes/no) as categorical covariates using negative binomial regression. Time at risk of experiencing an exacerbation was used as an offset variable in the model.

^b^It was assumed that the relative risks are equal to 1 due to lack of data (transition probabilities equal to BGF).

**Abbreviations:** BGF, budesonide/glycopyrronium/formoterol fumarate dihydrate; COPD, chronic obstructive pulmonary disease; FF/UMEC/VI, ﬂuticasone furoate/umeclidinium/vilanterol; NMA, network meta-analysis.

**Supplementary Table 4** Risk and relative risk of discontinuation by treatment

| Parameter | Monthly risk^a^ | | Relative risk vs BGF | |
| --- | --- | --- | --- | --- |
|  | BGF | Source | FF/UMEC/VI | Source |
| Initial risk of discontinuation | 1.85% | de Nigris 2022 (ETHOS) [2] | 0.90 | estimated using Paly 2022 (IMPACT)[5], de Nigris 2022[2] |

**Note:** ^a^The percentage of all patients discontinuing treatment after 52 weeks were presented by COPD state in the PAP analysis.

**Abbreviations:** BGF, budesonide/glycopyrronium/formoterol fumarate dihydrate; COPD, chronic obstructive pulmonary disease; FF/UMEC/VI, ﬂuticasone furoate/umeclidinium/vilanterol; PAP, payer analysis plan.

**Supplementary Table 5** Treatment-related AE incidence for BGF and relative risk for FF/UMEC/VI

| AE | Incidence^a^ | Relative risk (vs BGF)^b^ |
| --- | --- | --- |
|  | BGF | FF/UMEC/VI |
| Mild AEs | 18.30% | 1 |
| Moderate AEs | 27.10% | 1 |
| Severe AEs | 18.40% | 1 |

**Notes:** ^a^The incidences of AEs reported in the ETHOS clinical study report over 52 weeks were used in the model, and they reflect the proportions of patients that experienced treatment-related AEs over the trial duration.

^b^For FF/UMEC/VI, data from Bourdin 2021 was collected.[4]

**Abbreviations:** AE, adverse event; BGF, budesonide/glycopyrronium/formoterol fumarate dihydrate; FF/UMEC/VI, fluticasone furoate/umeclidinium/vilanterol.

**Supplementary Table 6** COPD severity level utility value

| COPD severity level utilities | ETHOS clinical trial EQ-5D-5L | |
| --- | --- | --- |
|  | All patients | Source |
| Moderate COPD utility | 0.79 | de Nigris 2022 (ETHOS)[2] |
| Severe COPD utility | 0.76 | de Nigris 2022 (ETHOS)[2] |
| Very severe COPD utility | 0.72 | de Nigris 2022 (ETHOS)[2] |

**Abbreviations:** COPD, chronic obstructive pulmonary disease; EQ-5D-5L, EuroQoL 5-dimension 5-level.

**Supplementary Table 7** Exacerbation and AE-related utility decrements used in the base case analysis

| Exacerbation events | QALY loss per event | Reference |
| --- | --- | --- |
| Moderate exacerbation disutility | 0.055 | Jackson et al 2024[6] |
| Severe exacerbation disutility | 0.090 | Jackson et al 2024[6] |
| AEs | Disutility | Reference |
| Mild AEs disutility | 0.000 | Assumption |
| Severe AEs disutility | 0.000 | Assumption |
| Very severe AEs disutility | 0.000 | Assumption |

**Abbreviations:** AE, adverse event; QALY, quality-adjusted life years.

**Supplementary Table 8** COPD management costs based on exacerbations

|  | No exacerbation history | | | Moderate exacerbation history | | | Severe exacerbation history | | |
| --- | --- | --- | --- | --- | --- | --- | --- | --- | --- |
|  | Moderate COPD | Severe COPD | Very severe COPD | Moderate COPD | Severe COPD | Very severe COPD | Moderate COPD | Severe COPD | Very severe COPD |
| Disease management costs per month, £ | 19.55 | 26.09 | 37.17 | 19.55 | 26.09 | 37.17 | 19.55 | 26.09 | 37.17 |

**Abbreviation:** COPD, chronic obstructive pulmonary disease.

**Supplementary Table 9** Drug acquisition costs[7]

| Treatments | Inhaler cost (£) | Number inhalations per inhaler | Dose per administration (number of inhalations) | Number of administrations per day | Cost per month (£) | Reference |
| --- | --- | --- | --- | --- | --- | --- |
| BGF | £44.50 | 120 | 2 | 2 | £45.15 | BNF 2024. Trixeo Aerosphere 5 micrograms/dose / 7.2 micrograms/dose / 160 micrograms/dose pressurised inhaler AstraZeneca UK Ltd. Date accessed: February 2024 |
| FF/UMEC/VI | £44.50 | 30 | 1 | 1 | £45.15 | BNF 2024 Trelegy Ellipta 92micrograms/dose / 55micrograms/dose / 22micrograms/dose dry powder inhaler GlaxoSmithKline UK Ltd. Date accessed: February 2024 |

**Abbreviations:** BGF, budesonide/glycopyrronium/formoterol fumarate dihydrate; BNF, British National Formulary; FF/UMEC/VI, ﬂuticasone furoate/umeclidinium/vilanterol.

**Supplementary Table 10** Number of rescue medication inhalations required per month per treatment by COPD severity levels and rescue medication acquisition cost[7]

| Treatments | Moderate COPD | Severe COPD | Very severe COPD | Reference |
| --- | --- | --- | --- | --- |
| BGF^a^ | 54.8 | 82.2 | 115.7 | AstraZeneca data on file (ETHOS PAP)[8] |
| FF/UMEC/VI | 54.8 | 82.2 | 115.7 | AstraZeneca data on file (ETHOS PAP)[8] |
| Treatment | Package cost (£) | Number of inhalations per inhaler | | Reference |
| Rescue medication: Ventolin 200 micrograms/dose Accuhaler | 1.99 | 60 | | BNF 2024 (date accessed February 2024) |

**Note:** ^a^Number of rescue medication inhalations required per month per treatment and by COPD severity level was calculated based on the difference between the mean baseline daily number of puffs per treatment and COPD state and the change in daily number of puffs per treatment from baseline over 52 weeks by COPD state, which is then multiplied with the number of days per month (365.25/12) [(“mean baseline daily number of puffs at baseline by baseline COPD state and treatment” + “change from baseline in mean baseline daily number of puffs by baseline COPD state and treatment”)*(365.25/12)] (eg, BGF for moderate COPD: (“mean baseline daily number of puffs at baseline for moderate COPD state and for BGF” + “change from baseline in mean baseline daily number of puffs for moderate COPD state and for BGF”)*(365.25/12) = (2.4 + (-0.6))*(365.25/12) = 54.8.

**Abbreviations:** BGF, budesonide/glycopyrronium/formoterol fumarate dihydrate; BNF, British National Formulary; COPD, chronic obstructive pulmonary disease; FF/UMEC/VI, ﬂuticasone furoate/umeclidinium/vilanterol; PAP, payer analysis plan.

**Supplementary Table 11** Treatment-related AE management costs[9]

| AEs | Unit costs (£) | Reference |
| --- | --- | --- |
| Mild AEs | 174.00 | National Schedule of Reference Costs Year: 2021-22 - Outpatient attendances - 300 General Medicine - Consultant-led unit cost |
| Severe AEs | 770.00 | National Schedule of Reference Costs Year: 2021-22 - Weighted average of the DZ65A-J COPD codes – Non-elective short stay |
| Very severe AEs | 2,995.00 | National Schedule of Reference Costs Year: 2021-22 - Weighted average of the DZ65A-K COPD codes - Non-elective long stay |

**Abbreviations:** AE, adverse event; COPD, chronic obstructive pulmonary disease.

**Supplementary Table 12** Subsequent treatment costs per month[7]

| Initial treatment | Subsequent treatment | Subsequent treatments costs |
| --- | --- | --- |
| BGF | Fixed-dose combination triple therapy plus romflumilast | £83.41^a^ |
| FF/UMEC/VI | Fixed-dose combination triple therapy plus romflumilast | £83.41^a^ |

**Note:** ^a^Roflumilast is given as 500 micrograms once daily. Each roflumilast package includes 30 tablets and costs £37.71. For the roflumilast treatment cost per month, first the cost per tablet was calculated by dividing the package cost by the number of tablets per package. Then the cost per tablet was multiplied with the number of tablets per administration and the number of administrations per day in line with the product’s summary of product characteristics and the number of days per month (365.25/12). [(“roflumilast package cost”/”number of tablets per roflumilast package”)*“number of roflumilast tablets per administration”*“number of administrations per day”*(365.25/12) = (£37.71/30)*1*1*(365.25/12) = £38.26]. Then the roflumilast monthly cost (£38.26) was added to the fixed-dose combination triple therapy monthly cost equal to £45.15.

**Abbreviations:** BGF, budesonide/glycopyrronium/formoterol fumarate dihydrate; FF/UMEC/VI, ﬂuticasone furoate/umeclidinium/vilanterol.

**Supplementary Table 13** Total costs for mortality waning

|  | 1-year^a^ | | 5-year | |
| --- | --- | --- | --- | --- |
| Outcome | BGF | FF/UMEC/VI | BGF | FF/UMEC/VI |
| Total costs, £ | 2,205 | 2,212 | 8,009 | 7,889 |
| Disease management costs, £ | 747 | 732 | 3,438 | 3,210 |
| Disease management costs (no exacerbation-related), £ | 285 | 284 | 1,312 | 1,245 |
| Exacerbation-related management costs, £ | 462 | 448 | 2,127 | 1,965 |
| Treatment-specific costs - before discontinuation, £ | 1,301 | 1,305 | 2,532 | 2,490 |
| Drug costs, £ | 483 | 487 | 1,647 | 1,607 |
| Rescue medication costs, £ | 26 | 26 | 94 | 91 |
| AE-related costs, £ | 792 | 792 | 792 | 792 |
| Subsequent treatment costs, £ | 103 | 93 | 1,299 | 1,161 |
| End of life costs, £ | 54 | 83 | 740 | 1,029 |

**Note:** ^a^Not applicable for 1-year results.

**Abbreviations:** AE, adverse event; BGF, budesonide/glycopyrronium/formoterol fumarate dihydrate; FF/UMEC/VI, ﬂuticasone furoate/umeclidinium/vilanterol.

**Supplementary Table 14** Different mortality distribution

| Outcome | BGF vs FF/UMEC/VI | |
| --- | --- | --- |
|  | 1-year | 5-year |
| ICUR (cost per QALY gained), £ | Dominant | 370.22 |
| ICER (cost per LY gained), £ | Dominant | 255.20 |
| Incremental costs per mortality avoided, £ | Dominant | 538.20 |
| INB based on WTP threshold £20 000 | 82.39 | 2,419.94 |
| Total incremental costs, £ | -23.06 | 45.64 |
| Incremental disease management costs, £ | 15.96 | 202.75 |
| Disease management costs (no exacerbation-related), £ | 1.29 | 56.19 |
| Exacerbation-related management costs, £ | 14.67 | 146.57 |
| Incremental treatment specific costs, £ | -3.24 | 29.26 |
| Drug costs, £ | -3.08 | 27.55 |
| Rescue medication costs, £ | -0.17 | 1.72 |
| Incremental subsequent treatment costs, £ | 10.33 | 128.12 |
| Incremental end-of-life costs, £ | -46.10 | -314.49 |
| Total incremental QALYs | 0.003 | 0.123 |
| Total incremental LYs | 0.005 | 0.179 |
| Total incremental exacerbations | 0.008 | 0.196 |
| Total incremental deaths avoided | 1.22% | 8% |

**Abbreviations:** BGF, budesonide/glycopyrronium/formoterol fumarate dihydrate; FF/UMEC/VI, ﬂuticasone furoate/umeclidinium/vilanterol; ICER, incremental cost-effectiveness ratio; ICUR, incremental cost-utility ratio; INB, incremental net benefit; LY, life year; QALY, quality-adjusted life year; WTP, willingness-to-pay.

**Supplementary Table 15** Setting during end-of-life

| Outcome | BGF vs FF/UMEC/VI | |
| --- | --- | --- |
|  | 1-year | 5-year |
| ICUR (cost per QALY gained), £ | 1,470.14 | 912.16 |
| ICER (cost per LY gained), £ | 916.91 | 631.17 |
| Incremental costs per mortality avoided, £ | 392.71 | 1,214.68 |
| INB based on WTP threshold £20 000 | 43.35 | 4,092.21 |
| Total incremental costs, £ | 3.44 | 195.56 |
| Incremental disease management costs, £ | 15.20 | 313.60 |
| Disease management costs (no exacerbation-related), £ | 1.02 | 97.90 |
| Exacerbation-related management costs, £ | 14.18 | 215.70 |
| Incremental treatment specific costs, £ | -3.65 | 73.66 |
| Drug costs, £ | -3.46 | 69.40 |
| Rescue medication costs, £ | -0.19 | 4.26 |
| Incremental subsequent treatment costs, £ | 10.15 | 181.90 |
| Incremental end-of-life costs, £ | -18.26 | -373.60 |
| Total incremental QALYs | 0.002 | 0.214 |
| Total incremental LYs | 0.004 | 0.310 |
| Total incremental exacerbations | 0.007 | 0.330 |
| Total incremental deaths avoided | 0.88% | 16.10% |

**Abbreviations:** BGF, budesonide/glycopyrronium/formoterol fumarate dihydrate; FF/UMEC/VI, ﬂuticasone furoate/umeclidinium/vilanterol; ICER, incremental cost-effectiveness ratio; ICUR, incremental cost-utility ratio; INB, incremental net benefit; LY, life year; QALY, quality-adjusted life year; WTP, willingness-to-pay.

**Supplementary figures:**

**Supplementary Figure 1** Linear waning applied in the model^a^

^a^For illustrative purposes only.

**Abbreviation:** BGF, budesonide/glycopyrronium/formoterol fumarate dihydrate.

**Supplementary Figure 2** Graphical representation of COPD health state mortality rates up to lifetime horizon A) moderate COPD and B) severe/very severe COPD

**Abbreviations:** BGF, budesonide/glycopyrronium/formoterol fumarate dihydrate; COPD, chronic obstructive pulmonary disease; FF/UMEC/VI, ﬂuticasone furoate/umeclidinium/vilanterol.

**References**

1. GOV.UK. Interactive health atlas of lung conditions in England (inhale): March 2023 update. Available from: <https://www.gov.uk/government/statistics/interactive-health-atlas-of-lung-conditions-in-england-inhale-march-2023-update/interactive-health-atlas-of-lung-conditions-in-england-inhale-march-2023-update>.

2. de Nigris E, Treharne C, Brighton N, Holmgren U, Walker A, Haughney J. Cost-effectiveness of triple therapy with Budesonide/Glycopyrronium/Formoterol fumarate dihydrate versus dual therapies in moderate-to-very severe chronic obstructive pulmonary disease: United Kingdom analysis using the ETHOS study. Int J Chron Obstruct Pulmon Dis. 2022;17:2987-3000.

3. Martinez FJ, Ferguson GT, Bourne E, et al. Budesonide/Glycopyrrolate/Formoterol fumarate metered dose inhaler improves exacerbation outcomes in patients with COPD without a recent exacerbation history: A subgroup analysis of KRONOS. Int J Chron Obstruct Pulmon Dis. 2021;16:179-89.

4. Bourdin A, Molinari N, Ferguson GT, et al. Efficacy and safety of Budesonide/Glycopyrronium/Formoterol fumarate versus other triple combinations in COPD: A systematic literature review and network meta-analysis. Adv Ther. 2021;38(6):3089-112.

5. Paly VF, Vallejo-Aparicio LA, Martin A, et al. Cost-effectiveness of once-daily single-inhaler COPD triple therapy in Spain: IMPACT trial. Int J Chron Obstruct Pulmon Dis. 2022;17:3097-109.

6. Jackson DJ, Jenkins M, de Nigris E, Purkayastha D, Patel M, Ouwens M. Associations between the EQ-5D-5L and exacerbations of chronic obstructive pulmonary disease in the ETHOS trial. Qual Life Res. 2024;33(4):1029-39.

7. British National Formulary. Pharmaceuticals prices 2018.

8. Le Rouzic O, Roche N, Cortot AB, et al. Defining the "Frequent Exacerbator" Phenotype in COPD: A Hypothesis-Free Approach. Chest. 2018;153(5):1106-15.

9. NHS England. Reference Cost Collection: National Schedule of Reference Costs, 2017-18 - NHS trusts and NHS foundation trusts. Available from: <https://www.england.nhs.uk/>.
